# Increased fucosylation has a pivotal role in invasive and metastatic properties of head and neck cancer stem cells

**DOI:** 10.18632/oncotarget.2698

**Published:** 2014-11-06

**Authors:** Vincenzo Desiderio, Petros Papagerakis, Virginia Tirino, Li Zheng, Margarite Matossian, Mark E. Prince, Francesca Paino, Luigi Mele, Federica Papaccio, Roberta Montella, Gianpaolo Papaccio, Silvana Papagerakis

**Affiliations:** ^1^ Department of Otolaryngology, Medical School, University of Michigan, Ann Arbor, MI, USA; ^2^ Department of Periodontics and Oral Medicine, School of Dentistry, University of Michigan, Ann Arbor, MI, USA; ^3^ Department of Experimental Medicine, Section of Biotechnology and Medical Histology and Embryology, Second University of Naples, Italy; ^4^ Department of Orthodontics and Pediatric Dentistry, School of Dentistry, University of Michigan, Ann Arbor, MI, USA; ^5^ Center for Organogenesis, Medical School, University of Michigan, Ann Arbor, MI, USA; ^6^ Department of Otolaryngology, Head and Neck Surgery, Medical School, University of Michigan, Ann Arbor, MI, USA; ^7^ Center for Computational Medicine and Bioinformatics, University of Michigan, Ann Arbor, MI, USA

**Keywords:** cancer stem cells, oral cancer, fucosylation, radiation, cisplatin

## Abstract

Oral squamous cell carcinoma (OSCC) is an aggressive malignancy with high mortality rates. Major challenges for OSCC management include development of resistance to therapy and early formation of distant metastases. Cancer stem cells (CSCs) have emerged as important players in both pathologic mechanisms. Increased fucosylation activity and increased expression of fucosylated polysaccharides, such as Sialyl Lewis X (SLe^x^), are associated with invasion and metastasis. However, the role of fucosylation in CSCs has not been elucidated yet. We used the spheroid culture technique to obtain a CSC-enriched population and compared orospheres with adherent cells. We found that orospheres expressed markers of CSCs and metastasis at higher levels, were more invasive and tumorigenic, and were more resistant to cisplatin/radiation than adherent counterparts. We found fucosyltransferases FUT3 and FUT6 highly up-regulated, increased SLe^x^ expression and increased adhesion by shear flow assays in orospheres. Inhibition of fucosylation negatively affected orospheres formation and invasion of oral CSCs. These results confirm that orospheres are enriched in CSCs and that fucosylation is of paramount importance for CSC invasion. In addition, SLe^x^ may play a key role in CSC metastasis. Thus, inhibition of fucosylation may be used to block CSCs and metastatic spread.

## INTRODUCTION

Head and neck (H&N) squamous cell carcinoma (HNSCC) is one of the world's top ten most common cancers: in fact, it is ranked the 8^th^ and 13^th^ most common malignancy, respectively, for males and females [1-3]. Unfortunately, current treatments for HNSCC can be traumatic and disfiguring, drastically affecting quality of life [4, 5]. Management of HNSCC includes surgical resection and/or a combination of chemo- and radio-therapy [1, 6]. Despite these treatments, the prognosis of HNSCC remains poor due to late diagnosis, high rates of primary-site recurrence, and common metastases to loco-regional lymph nodes [2, 6, 7].

Oral squamous cell carcinoma (OSCC), which includes epithelial neoplasms of the oral cavity and oropharynx, accounts for the majority of HNSCCs [8] and causes cancer-related mortality with an estimated >275,000 new cases and >120,000 deaths per year [9]. Despite the numerous advances in diagnosis and treatment of oral cancer, mortality and morbidity rates for OSCC are exceedingly high: the five-year survival rate of stage I cancer including the various sub-sites, such as borders of the tongue, floor of the mouth, cheek, and gums, is approximately 80%, while the five-year survival rate of patients with advanced disease (stages III/IV) is approximately 20%. Worldwide, about 50% of OSCC patients are diagnosed with advanced disease and the available treatment modalities are still limited. Therefore, novel treatment options and diagnostic tools are needed to improve disease outcome.

Cancer stem cells (CSCs) are a small subpopulation of self-sustaining cancer cells with the ability to form the heterogeneous cell lineages that compose the tumor [10]. CSCs are characterized by three main features: i) potent tumor initiation; ii) self-renewal *in vivo* (observed practically via re-growth of phenotypically indistinguishable tumors following serial transplantation of re-isolated CSCs in secondary and tertiary recipients); and iii) cell differentiation capacity, allowing them to give rise to a heterogeneous progeny representing a phenocopy of the original tumor [11]. CSCs have been proposed to be responsible for the aggressive behavior of several cancer types via the appropriation of the molecular machinery of homing and mobilization involved in tumor invasion and metastasis [12].

There are many methodologies to detect, isolate, and characterize CSCs from tumors: the main methods are cell sorting based on stemness marker expression, side population profiling, and formation of floating spheres [13-15]. Sphere formation *in vitro* allows selection of CSC-rich populations, and this method is particularly useful when specific CSC makers have not been well defined, as is the case for most cancer types [16]. CSCs have been identified in many solid tumors, including breast [17], lung [18], colon [19], prostate [20], ovary [21], brain cancer [22], and sarcoma [23]; in H&N cancer, the existence of CSCs was first assessed using CD44 as a stem cell marker [24].

Fucosyltransferases (FUTs) are a family of Golgi-apparatus enzymes that transfer L-fucose from GDP-fucose to a glycoside or a peptide. According to the fucosylation site, FUTs are classified into alpha-1,2 (FUT1 and FUT2), alpha-1,3/4 (FUT3, FUT4, FUT5, FUT6, FUT7, and FUT9), and alpha-1,6 (FUT8) [25]. In mammals, fucosylated glycans are involved in cell adhesion during development [26, 27], the inflammatory response, and leukocyte trafficking [28, 29]. Great attention has been paid to FUTs and their inhibitors over the past 20 years due to the fact that addition of L-fucose is involved in a series of diseases, including cancer and metastatic spread [30-33]. Sialyl Lewis X (SLe^x^) is a cell-surface tetrasaccharide carbohydrate involved in many recognition processes. It is synthesized in the Golgi compartment by different glycosyltransferases, with the final step involving the transfer of L-fucose to N-acetylglucosamine by alpha-1,3-FUT3/5/6/7, depending on the cell type [34].

In this study, we extensively compared orospheres with their adherent cell counterpart in terms of gene expression, stem cell and metastasis marker profile, cell adhesion and invasion, potential to form tumors in an animal model, and resistance to drugs and radiation. Moreover, we show that inhibition of fucosylation affects the orosphere formation and invasion ability of CSCs.

## RESULTS

### Orospheres formation

The ability to grow in suspension in serum-free medium was investigated with a tumor-initiating cell-selection method. OSCC orospheres were clearly observed already after 24 h in serum-free medium. After 7 days of culture, orospheres were seeded onto standard plates with 10% FBS. Cells migrated from the spheres within a few hours and adhered to the bottom of the flasks, assuming their original shape.

### Orospheres have protein and RNA expression typical of CSCs and increased propensity to grow *in vivo*

Orospheres and corresponding adherent cells of two OSCC lines (UMSCC14B and UMSCC103) were assessed for expression of CD44, CD29, CD56, sialyl Lewis^X^ (SLe^X^), sialyl Lewis A (SLa) and Lewis Y (Ly) as well as for aldehyde dehydrogenase (ALDH) activity. CD44 and ALDH activity are the most common markers of CSCs in OSCC [24, 35]; CD29 is a β1-integrin involved in invasion and metastasis that has been used as a CSC marker for different cancers [36]; and CD56 (N-CAM), which we describe here for the first time in oral cancer, has been correlated with invasion and distant metastasis in many cancers [37-39].

**Figure 1:**
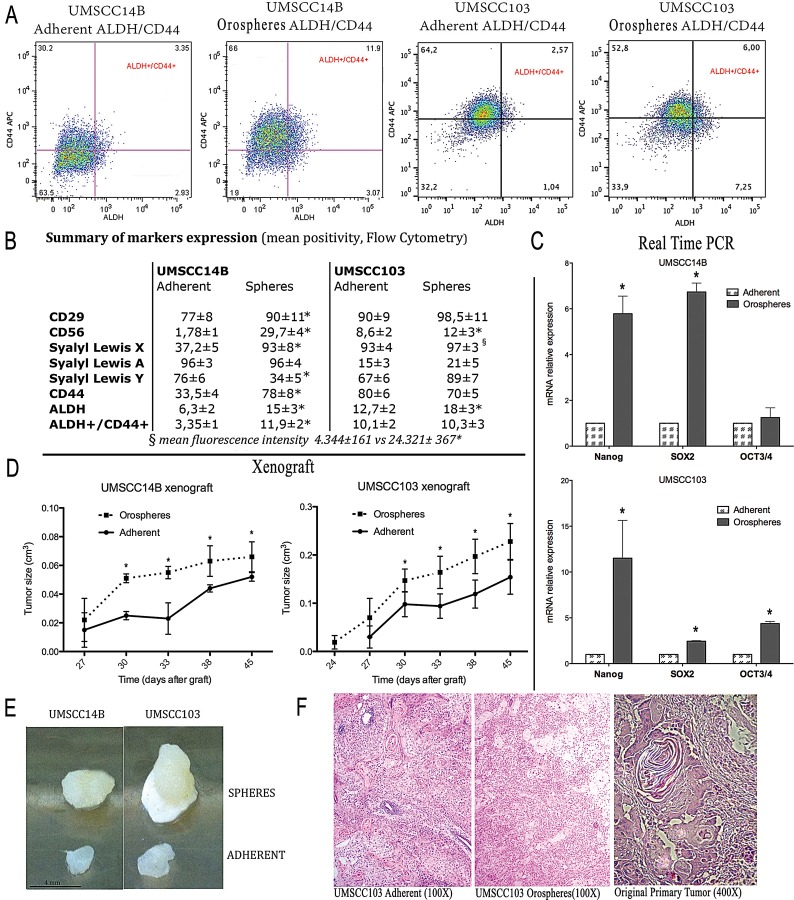
Cancer stem cell characterization A. Flow cytometry analysis of CD44 and ALDH activity. The CD44^+^/ALDH^+^ subpopulation is larger in orospheres in both UMSCC14B and UMSCC103 cell lines. B. Summary of marker expression in orospheres and adherent cells. *P<0.05 C. Real time PCR for the most common stem cell-related markers (OCT3/4, SOX-2, NANOG). All stem cell-related markers are remarkably more expressed on orospheres. *P<0.05. D. Growth curve of orospheres and adherent cells implanted in immunocompromised mice. In both cell lines, the orospheres show faster growth, resulting in larger tumors at the time of sacrifice. E. Examples of explanted tumors for both cell lines. Orospheres produce remarkably larger tumors. Scale bar=4mm. F. H&E staining on sections of explanted and original primary tumors. Orospheres and adherent cells recapitulate the architecture of the original tumor. Original magnification: 100X (xenograft); 400X (original primary tumor).

Cytometric analyses revealed that orospheres were remarkably more positive for ALDH activity than their adherent counterpart. In UMSCC14B cells, CD44 was more expressed on orospheres, while in the UMSCC103 cell line CD44 was more expressed by adherent cells. Nevertheless, in both cell lines, CD44–ALDH double-positivity was remarkably higher in orospheres than corresponding adherent cells (Fig. 1A).

Similarly, CD29, CD56, and SLe^x^ were consistently higher on orospheres than adherent cells (Fig1B). SLa was highly expressed by UMSCC14B but only slightly expressed by UMSCC103 cell lines. No variations were observed between orospheres and adherent cells in both cases. Ly had a different pattern, being more expressed by orospheres in the UMSCC14B cell line, but more expressed by adherent cells in the UMSCC103 cell line. Neither SLa nor Ly seemed to be associated with the orospheres culture condition (Fig. 1B).

RNA from orospheres and adherent cells from both cell lines was extracted and PCR performed for genes related to stemness and metastasis. ALDH RNA expression was higher in orospheres of both cells lines, showing that orospheres not only have higher enzyme activity but that they also produce more ALDH. N-CAM RNA expression levels were also consistently higher in orospheres (Supplementary Table 1), as assessed by the cytometric analysis. Furthermore, the RNAs of VEGF, CXCR2, MMP10, PAR6, and FXYD5 (dysadherin), well-known markers associated with metastasis and poor prognosis in different cancers, including OSSC [40-43], were all up-regulated in orospheres (Supplementary Table 1), as were the RNAs of commonly recognized markers of normal stem cells and CSCs, such as SOX-2, OCT3/4, and NANOG (Fig. 1C).

To evaluate tumorigenic potential, orospheres and adherent cells were injected into the flanks of NOD/SCID immunodeficient mice. Tumor growth was measured every three days after tumors were first detected. 24-27 days after implantation, both orospheres and adherent cells were found to generate tumors in all cases, but although the same number of cells were injected, tumor size and growth of orospheres was significantly greater than that of adherent cells (p<0.05) (Fig. 1D and 1E). Hematoxylin and eosin staining revealed that the xerographs reconstituted the characteristics of the original primary tumor architecture (Fig. 1F).

### Orospheres are resistant to Cisplatin and radiation treatment

**Figure 2:**
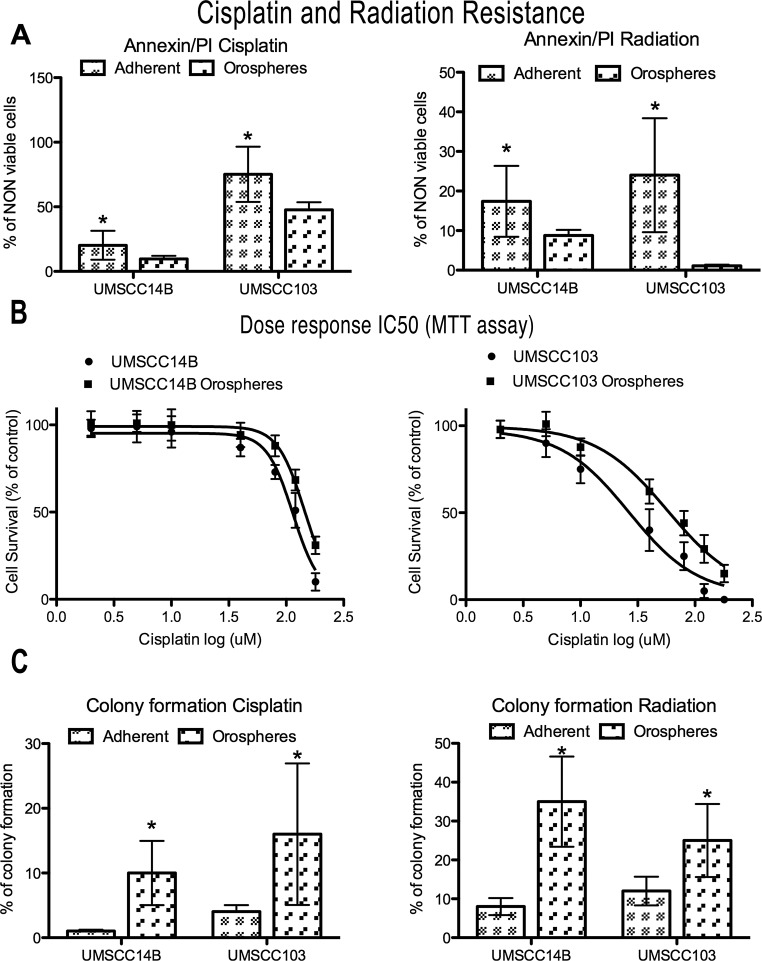
Cisplatin and radiation resistance A. Graphs of non-viable (apoptotic+necrotic) cells measured by Annexin V/PI staining after cisplatin (after 24 hours) or radiation (after 48 hours) treatment. Orospheres are more resistant to both treatments. *P<0.05. B. Dose-response curves for IC50 calculation (UMSCC14B adherent cells, 114 (95% CI 104-126); UMSCC14B orospheres, 144 (95% CI 134-155); UMSCC103 adherent cells, 26.5 (95% CI 18-39); UMSCC103 orospheres, 58 (95% CI 47-72). C. Colony formation was measured 15 days after cisplatin or radiation treatment. Orospheres form significantly more colonies than adherent cells. *P<0.05.

We performed a preliminarily dose-response evaluation of the growth inhibition induced by either cisplatin or radiation on adherent cell lines after 24h of treatment (Supplementary Figure 1). On the basis of the results, we used for all the subsequent experiments the concentrations that caused at least 20% cell death. Therefore, the cell lines were treated with 80μM cisplatin and cell death analyzed after 24 hours. In both cell lines, orospheres had greater resistance to cisplatin-induced death than the corresponding adherent cells (Fig. 2A and Supplementary Table 2). In particular, the UMSCC103 cell line had higher sensitivity to cisplatin treatment, both as orospheres and adherent cells. In addition, IC50 was calculated from the dose-response curve assessed with a MTT assay (Fig.2B). For both cell lines, IC50 of orospheres was significantly higher than for adherent cells. IC50 values were: 114 (95% CI 104-126) for UMSCC14B adherent cells, 144 (95% CI 134-155) for UMSCC14B orospheres, 26.5 (95% CI 18-39) for UMSCC103 adherent cells, and 58 (95% CI 47-72) for UMSCC103 orospheres.

We also assessed radiation resistance. Even for this death stimulus, orospheres were more resistant than corresponding adherent cells (Fig. 2A). In both cell lines, a slight increase of necrosis was also found (Supplementary Fig. 2 and table 2). The findings were confirmed by colony forming assay (Fig. 2C and Supplementary Table 2): while adherent cells formed no or few colonies, orospheres were consistently able to form visible colonies.

### Orospheres are more invasive and show increased fucosylation

**Figure 3:**
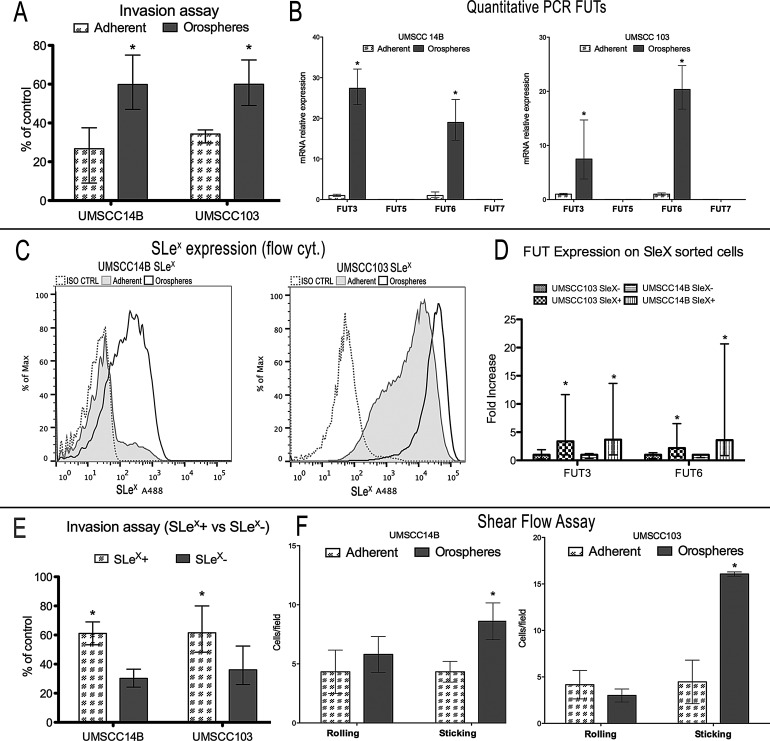
Fucosylation and SLe expression - functional assays A. Invasion assay of orospheres vs adherent cells. Orospheres are significantly more invasive than adherent cells. B. Quantitative mRNA expression of FUTs. In both cell lines, FUT5 and FUT7 are not expressed, while FUT3 and FUT6 are more expressed in orospheres. C. Sialyl Lewis X (SLe^x^) expression on orospheres and adherent cells (flow cytometry). Orospheres express a higher level of SLe^x^ as compared with their adherent counterparts (UMSCC14B: 93% for orospheres vs 27.2 for adherent cells; UMSCC103: 97% for orospheres vs 93% for adherent cells). Moreover, the mean fluorescence intensity for UMSCC103 is much higher than that of adherent cells, with most of the cells after the 4^th^ decade of fluorescence intensity. D. qPCR for FUT 3/6 expression on SLe^x^ sorted cells. SLe^x+^ cells have significantly increased FUTs expression. E. Invasion assay for SLe^x+^ vs SLe^x−^. SLe^x+^ cells are significantly more invasive than SLe^x−^ cells. F. Shear flow assay. Graphs of percentages of firmly adherent (sticking) and rolling cells. The rate of firmly adherent (sticking) cells is higher for orospheres in both cell lines, while there is no significant difference in rolling cells. Moreover, the mean velocity of rolling cells is much lower for orosphere-derived cells than for adherent cells. *P<0.05.

The invasive ability of cells was then assessed with a Matrigel invasion chamber assay. Orospheres derived from the cell lines were significantly more invasive than adherent cells: UMSCC14B orospheres-derived cells had a 2.4-fold increase, while those derived from UMSCC103 orospheres had a 1.75-fold increased ability to migrate (Fig. 3A). Moreover, expression of FUT3 and FUT6 mRNA was significantly higher in orospheres (Fig. 3B), but FUT5 and FUT7 were not expressed in either adherent cells or orospheres (Fig. 3B). In addition, orospheres of both cell lines had a strong increase in the fucosylated carbohydrate SLe^x^ (Fig 3C). In order to further investigate a possible relation between FUT and SLe^x^ expression, we sorted cells for surface SLe^x^, extracted the RNA, and performed qPCR for FUTs 3/6 expression. In both cell lines, the SLe^x+^ subpopulation had a significantly higher expression of FUTs (Fig 3D). Consequently, we found that SLe^x+^ cells had a significantly greater invasive ability than SLe^x−^ cells. (Fig. 3E).

Shear flow is a functional analysis assessing the ability of cells to roll and stick on E-selectin-expressing endothelium. Rolling and adhesion to E-selectin-expressing endothelial cells is a key step for metastasis initiation. Orospheres-derived cells showed a significantly greater ability to adhere to E-selectin-expressing CHO cells than did adherent cells (Fig. 3F). In UMSCC14B and UMSCC103 cells, adhesion of orosphere-derived cells was respectively 2-fold and 3.58-fold greater than their adherent counterpart. Rolling was not significantly different in the former; in the latter, orospheres rolled at slightly lower percentages than adherent cells, but the difference was not significant (Fig. 3F).

Of note, the maximum velocity calculated for UMSCC103 orospheres-derived cells (31.83 μm/s) was about half that of the adherent cells (60.82 μm/s), indicating that orospheres cells are more adherent to the monolayer. This correlation was found also for the UMSCC14B cell line.

### FUT inhibition reduces orospheres formation

**Figure 4:**
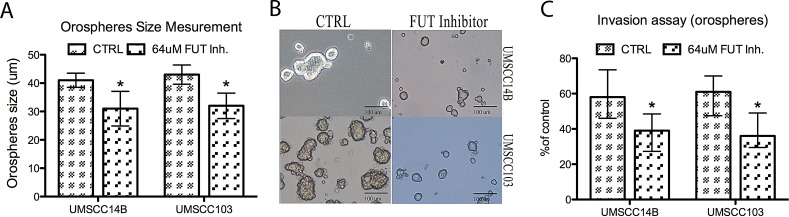
FUT inhibition A. Orosphere size in cells treated with 2F-peracetyl-fucose. Treated cells of both lines produced significantly smaller sized orospheres. B. Image of orospheres from 2F-peracetyl-fucose-treated and -untreated cells. C. Invasion assay of orospheres treated and untreated with 2F-peracetyl-fucose. Treated cells had significantly reduced invasion ability.

In order to evaluate the effect of fucosylation on orospheres formation, we incubated adherent cells with the specific inhibitor 2F-peracetyl-fucose for 72 hours prior to detaching the cells for orospheres formation. Inhibition of fucosylation with 2F-peracetyl-fucose resulted in the formation of fewer and smaller orospheres formation (Fig. 4A and B). Moreover, treatment significantly reduced invasion ability of orospheres (Fig. 4C): UMSCC14B cells had a 1.49-fold decrease, while UMSCC103 cells had a 1.7-fold decrease after treatment.

## DISCUSSION

Prince el al. first described CSCs in H&N cancer as a subpopulation of CD44^+^/CD24^−/low^ cells [24]. Later, Clay et al. used ALDH1 activity to additionally characterize the stem-like subpopulation in H&N cancers [35]. Recently, floating spheres have also been used to elucidate the roles of CSCs in H&N cancer [44, 45].

To date, there is no available therapy targeting CSCs directly. A detailed characterization of CSCs and the discovery of new mechanisms involved in the metastatic process, stem-like feature acquisition, and drug resistance is necessary for the identification of more-effective strategies against this aggressive and resistant subset of cells. Increased fucosylation has been associated with metastatic and invasive properties of cancer cells [46-48]; moreover, inhibition of fucosylation has been successfully used against tumor growth and metastasis *in vitro* and *in vivo* [49, 50]. In this light, our aim was to understand the role of fucosylation in CSCs-mediated invasion and metastasis in OSCC.

We firstly characterized H&N-derived CSCs in terms of marker expression, tumorigenic potential, and metastatic and invasive ability. Both cell lines studied were able to grow as orospheres that could be passaged for at least three times. Orospheres and adherent cells were analyzed comparatively for expression of several markers, including ALDH activity and CD44. It is generally accepted that ALDH activity and CD44 expression are reliable markers for CSC identification in carcinomas from the head and neck areas [24, 35, 51]. In our study, orospheres from both cell lines had increased ALDH activity compared with adherent counterparts. On the other hand, CD44 expression was higher in UMSCC14B orospheres than in adherent cells, differently to the UMSCC103 cell line, in which CD44 was more expressed on adherent cells. Nevertheless, the percentage of double-positive cells for ALDH activity and CD44 expression was higher in orospheres than in adherent cells for UMSCC14B and UMSCC103 cell lines. Previous studies suggested that CD44 is also highly expressed in normal oral epithelium, similar to the expression detected in OSCC [52], while various CD44 variant isoforms that arise from alternative exon splicing are exclusively expressed in tumors, where they were significantly associated with advanced primary tumor stage, metastasis, treatment failure, and reduced disease-free survival [53, 54]. For these reasons, CD44 should be used in combination with other markers for the reliable identification of CSCs.

Furthermore, orospheres had a pattern of gene expression typical of stem cells, characterized by up-regulation of genes such as *OCT4*, *NANOG,* and *SOX2*. These findings were reinforced by up-regulation of other genes related not only to stemness but also to tumor progression and negative outcome, including *NCAM* and *PAR-6* [42]. Interestingly, to our knowledge, CD56 (NCAM) has not been described before in OSCC-derived CSCs. NCAM is an homophilic binding glycoprotein expressed on the surface of various cells (e.g. neurons, glia, skeletal muscle, and natural killer cells), which has been correlated to invasion and distant metastasis in many human cancers, including small cell lung cancer, thyroid cancer, hepatocellular carcinomas, and glioma [37-39]. In order to better understand the potential links between CSCs and metastasis formation, we performed PCR arrays for genes related to metastasis. We found that orospheres overexpressed several genes, such as *VEGF*, *FXYD5* (dysadherin), *CXCR2*, and *MMP10,* that have been strongly correlated with invasion and metastasis in many cancers, including H&N carcinomas [40, 41, 43].

Two of the most important characteristics of CSCs are the ability to generate tumors in immunocompromised mice [19, 44] and resistance to conventional therapy [44, 55]. Here, we have demonstrated that tumors originating from orospheres grow faster and bigger than those originating from adherent cells, confirming the capacity of orospheres to initiate and sustain tumor growth. Moreover, cells derived from orospheres were significantly more resistant to cisplatin and radiation treatment than adherent cells. These results strongly suggest that orospheres are enriched in CSCs and that they represent a highly reproducible model for studying CSCs in OSCC.

Sialyl Lewis X is an E-selectin ligand with a carbohydrate structure that is constitutively expressed on granulocytes and monocytes, mediating inflammatory extravasation of these cells [56]. We found that SLe^x^ expression was strongly associated with orospheres, confirming our recent preliminary report [57]. In different cancers, SLe^x^ expression has been related to poor prognosis and metastasis, suggesting that it may be involved in cancer cell extravasation [58-61]. Consistently, we found increased expression of FUT3 and FUT6 (involved in SLe^x^ synthesis) in orospheres concurrently to increased levels of SLe^x^. In our model, other FUT3/6 products, such as SLa and Sly, were not associated with the orosphere culture condition. The expression pattern of these two Lewis family saccharides is discordant with the increase in FUT3/6 that we found in orospheres, suggesting that other enzymes may be responsible for their regulation. At the same time, sorted SLe^x+^ adherent cells expressed significantly higher levels of FUT3/6 than did SLe^x−^ cells. In addition a causal correlation between FUT3/6 and SLe^x^ levels has been showed in other models[34, 62-65]

Taken together, these findings strongly suggest that increased expression of FUT3/6 is associated with increased SLe^x^ production. An invasion assay indicated that SLe^x+^ cells (with high FUTs expression) levels were significantly more invasive than SLe^x−^ cells (with low FUTs), suggestive of an association between FUTs expression and invasive phenotype.

Moreover, shear flow assays, mimicking the potential interaction between tumor cells and endothelium, showed that orospheres are remarkably more able to adhere to E-selectin-expressing cell layer, a feature that is of paramount importance for distant metastasis formation [60, 66]. We did not find a significant difference in the number of rolling cells, but we did find a consistent and substantial difference in firmly adherent cell number and in the mean velocity of rolling cells. Thus, orospheres-derived CSCs have greater ability to bind to E-selectin. SLe^x^ is the main E-selectin ligand, and we found it to be overexpressed on orospheres-derived cells with increased FUT3/6 expression. 2F-peracetyl-fucose is the only commercially available inhibitor of fucosylation [67]. We show that inhibition of fucosylation negatively affects orospheres formation, producing smaller-sized spheroids. Sphere formation is widely used to enrich the CSC population, and is recognized as a standard tool to assess and confirm self-renewal in stem-like cells. Interestingly, inhibition of fucosylation also affected invasion ability of sphere-derived CSCs. Even though *in vitro* migration assays are not comprehensive methods for broad characterization of the invasive process, they have been extensively used and correlated to cancer invasion *in vivo* [63, 64]. Moreover, inhibition of fucosylation affects E-selectin binding and cell extravasation [49], and Cheng et al. [68] showed that FUT family members, including FUT6, are involved in multidrug resistance in hepatocellular carcinomas. Based on this previous data and on the findings of this study, we hypothesize that increased fucosylation may be a mechanism used by CSCs to acquire not only invasive and metastatic features, but also resistance to conventional therapy.

## CONCLUSIONS

Our study demonstrates the following: (i) orospheres are enriched in CSCs, express higher levels of metastatic markers, and are more tumorigenic than adherent counterparts; and (ii) fucosylation is of paramount importance in the invasion and metastatic process of CSCs. Among fucosylated saccharides, Sialyl Lewis X is a strong candidate in the acquisition of a FUT-associated invasive phenotype. Thus, inhibition of fucosylation may represent an active therapeutic tool against cancer stem cells and metastatic spreading.

## METHODS

### Ethics Statement

The investigation was conducted in accordance with the ethical standards and according to the Declaration of Helsinki and according to national and international guidelines and has been approved by the authors' institutional review board.

### Cell culture and orospheres formation

OSCC cell lines (UMSCC14B, UMSCC103), used in this study were established at the University of Michigan under a protocol approved by the Institutional Review Board Office in accordance with the university's regulations [69, 70]. Cells were cultured in DMEM (Gibco, NY, USA) supplemented with 2 mM glutamine, 100 IU/ml penicillin, 100 μg/ml streptomycin (Invitrogen, Carlsbad, CA), and 10% heat-inactivated fetal bovine serum (FBS) (Invitrogen Life Technologies, NY, USA) at 37°C in a humidified atmosphere under 5% CO_2_. Cells were passaged at a split ratio of 1:3–1:6. The cell lines used in this study were negative in periodic monitoring for mycoplasma. The cell lines were also genotyped to rule out cross-contamination and their morphology was regularly examined.

For orosphere formation, cells were plated at a density of 3 × 10^5^ cells in 25 cm^2^ ultra-low-attachment flasks (Corning, NY, USA) in DMEM/F12 supplemented with B27 and N2 supplement (Gibco, NY, USA), 100 IU/ml penicillin, and 100 μg/ml streptomycin (Invitrogen, Carlsbad, CA). Human fibroblast growth factor (FGF, 20ng/ml) and epidermal growth factor (EGF, 20ng/ml) (both from Sigma Aldrich, MO, USA) were added every other day. After 48–72 hours, orospheres were enzymatically dissociated and re-cultured in the same condition to form second- and then third-passage orospheres, which were used for experiments. Inhibition of fucosylation was obtained as described by Rillahan et al. [67] using the fucosyltransferase inhibitor 2F-Peracetyl-Fucose (Millipore Calbiochem, Merck KGaA, Darmstadt, Germany) at 64 μM for 72 hours prior to performing experiments. To assess effect of 2F-Peracetyl-Fucose on orosphere formation, the number and size of orospheres were measured. At least 50 spheres for each sample were measured, reporting the diameter as mean with 95% confidence interval. 2F-Peracetyl-Fucose did not affect viability at the concentration used (Supplementary Figure 3).

### Phenotypic characterization and ALDH activity assay of orospheres vs adherent cells

For flow cytometry, adherent cells were detached with trypsin/versene dissociation medium (Gibco, NY, USA). Briefly, the culture medium was discharged, cells were washed in PBS, and dissociation medium added and kept until cells detached from flasks. For dissociation of spheres, spheres were collected by centrifugation, washed with PBS (Gibco, NY, USA), and then 1 ml of dissociation medium added until the orospheres were completely dissociated. Cells were re-suspended at 1×10^6^/ml and incubated with primary antibody for 30 minutes on ice in the dark. Secondary antibodies, when needed, were added after a PBS wash for 30 minutes in the same conditions. Primary antibodies were PE-CD24, PE/Cy5-CD29, FITC-CD56 (N-CAM), not conjugated CD15s (SleX, mouse IgM), and APC-CD44 (BD, CA, USA). SLa (sialyl Lewis A, mouse IgG) and Ly (Lewis Y, mouse IgM) were not conjugated (Abcam, Cambridge, UK). Alexa488-conjugated secondary anti-mouse IgM and IgG antibodies were purchased from Life Technologies (NY, USA).

The Aldefluor kit (STEMCELL Technologies, BC, Canada) was used to identify cell populations with high aldehyde dehydrogenase (ALDH) activity. Briefly, 10^6^ harvested cells were resuspended in Aldefluor assay buffer containing ALDH substrate, as recommended by the producer. As a negative control for all samples, an aliquot of “Aldefluor-exposed” cells was immediately quenched with a specific ALDH inhibitor, diethylaminobenzaldehyde (DEAB). After incubation for 30 min at 4°C and following centrifugation, the cells were resuspended in cold Aldefluor buffer, and stained with 1 μg/ml propidium iodide (PI) (Sigma Aldrich, MO, USA) to discriminate viable from dead cells. Aldefluor staining was detected in a green fluorescence channel. Samples treated with the inhibitor DEAB (+DEAB) were used as controls to set the gates defining the ALDH^+^ region. Flow cytometry analyses and sorting were performed at the University of Michigan Flow Core using a FACS ARIA III (Becton Dickinson), a MoFlow Astrios (Beckman Coulter, Inc), a FACS Canto III (Becton Dickinson), or a MacsQuant (Miltenyi Biotec). All data were analyzed by Flow-Jo software (Tree Star, Inc).

### RNA isolation, qRT-PCR, and PCR arrays

Total RNA was isolated by using TRIzol (Invitrogen, NY, USA). RNA (2 μg) was reverse transcribed with TaqMan reverse transcription reagents (Applied Biosystems, Branchbury, NJ, USA), following the manufacturer's recommendations. The resulting cDNA was then amplified by real time RT-PCR (qRT-PCR) using AmpliTaq Gold DNA Polymerase (Applied Biosystems). The RT-PCR products were subcloned into pGEM-T Easy vector (Promega, Madison, WI) and confirmed by sequencing. For RNA quantification, qRT-PCR amplifications were performed at 95°C for 30 s, 60°C for 30 s, and 72°C for 30 s using specific primers for the house-keeping gene β-actin (Actb). The PCR primers sequences are given in Supplementary Table 1. The relative expression levels for each gene were calculated based on the expression levels of Actb and the differences are presented in graphs using the 2-ΔΔCT method. P-values were calculated using two-sample t-test.

PCR arrays were purchased from Qiagen (QIAGEN, Valencia, CA). The arrays and data analyses were performed at University of Michigan's DNA core.

### Xenograft formation in NOD/SCID mice

Adherent cells and orospheres were enzymatically dissociated to obtain single-cell suspensions, diluted to 5×10^4^/50μl in PBS, mixed with 50 μl of Matrigel (Becton Dickinson, CA, USA), and injected subcutaneously in 6-week-old female NOD/SCID mice (Harlan Italy, Milan, Italy). Each mouse received an adherent cell injection in the left flank and a spheroid-cell injection in the right flank. Mice were monitored every 3 days for the appearance of subcutaneous tumors. Tumor size was measured weekly with calipers. Tumor volume (V) was calculated as follows: V = W^2^×L×0.5, where W and L were tumor width and length, respectively. After 45 days, mice were sacrificed, and the tumor tissue was collected and fixed in buffered formalin. Hematoxylin and eosin (H&E) stain was performed to determine tumor histology. All procedures and experiments involving animals were approved and conducted according to the regulations of the Animal Ethic Committee of the Second University of Naples.

### *In vitro* treatment with cisplatin

Cisplatin (cis-diammineplatinum (II) dischloride, DDP) (Sigma-Aldrich, St. Louis, MO, USA) was dissolved in sterile 0.9% NaCl to achieve a stock concentration of 5 mM. Cells were plated 3–5−10^5^ in 25cm^2^ flasks, left to grow for 24 hours, and then treated. To establish cisplatin dosage for further experiments, cytotoxic curves were performed with Annexin V/PI staining kit on adherent cells at the concentrations of 2 μM, 5 μM, 20 μM, 40 μM, 80μM, and 100μM for 24 hours (Supplementary Figure 1). The cisplatin dose was chosen in order to produce at least 20% reduction in viability. Cells were then treated with cisplatin 80μM for 24 hours in the subsequent experiment.

### *In vitro* treatment with radiation

Cells are irradiated at the Experimental Irradiation Core of the Comprehensive Cancer Center, University of Michigan. 250kV X-ray radiation (Philips RT250, Kimtron Medical) was delivered at a dose rate of approximately 2 Gy/min. Dosimetry was carried out with an electrometer system directly traceable to a National Institute of Standards and Technology calibration. Both cell lines considered were given 6 Gy.

### IC50 calculation and MTT assay

In order to calculate IC50, a dose-response curve was obtained by treating both cell lines with cisplatin, either in adherent or sphere culture conditions. Cells were plated at 1×10^4^ per well in 96-well plates before treatment with the following concentrations of cisplatin: 2 μM, 5 μM, 20 μM, 40 μM, 80μM, 120 μM, and 180 μM. After 24 h of treatment, MTT solution (1 mg/mL in PBS) was added to each well. The plates were then incubated at 37 °C for 4 h, and reduced purple-blue MTT formazan crystals were solubilized by adding 200 μL of DMSO to each well. The absorbance was measured at 595 nm using a microplate ELISA reader, with DMSO used as the blank. IC50 was calculated using Prism GraphPad Sofware (GraphPad Software, La Jolla, CA USA).

### Colony Forming Assay

Orospheres and adherent cells received either 80 μM cisplatin for 24 hours or 6 Gy. Cells were then washed twice with PBS, adherent cell detached, and orospheres disaggregated using trypsin/EDTA for 5 minutes. Cells were then seeded at 250/cm^2^. Untreated cells for both adherent cultures and orospheres were used as control and to assess plating efficiency. Cultures were observed for 7–14 days (depending on growth rate differences between cell lines) to allow untreated cells to reach >50 cells/colony. Colonies were then fixed and stained with crystal violet in 20% methanol. Plating efficiency (PE) was calculated by dividing the number of colonies formed in the no treatment group by the number of cells seeded (PE=# of colonies formed/# of cells seeded). Survival fraction (SF) was determined by colonies formed after treatment divided by the number of cells seeded multiplied by the plating efficiency (SF=# colonies formed/# of cells seeded x PE). All experiments were done in triplicate.

### Shear flow assay

Regular and E-selectin stably transfected Chinese Hamster Ovarian cells (CHO and E-sel-CHO cells were a gift from Dr Lloyd Stoolman, Pathology Department, University of Michigan), that grow as monolayers in culture, were maintained in MEM-α media (20% FBS, 1X NEAA, 1X pen-strep). CHO cells were counted using a Beckman Coulter Counter and then incubated with the fluorescent marker CFSE (5-(and -6)-carboxyfluorescein diacetate succinimidyl ester) diluted with PBS (25 μM) for 30 minutes. The cells were then centrifuged for 5 minutes at 1300 rpm, re-suspended at 1 × 10^6^ cell/ml and incubated for 30 minutes in DMEM media (10% FBS, 1X NEAA, 1X pen-strep). The tumor cells were perfused over the CHO monolayer at 1.4 dynes/cm^2^ using a Harvard Apparatus. The shear force was calculated using the equation y=6Qμ/bh2, where y is the shear force in dynes/cm^2^, Q is the flow rate in mL/sec, μ is the apparent viscosity (which is 0.01 poise for water at 32°C), and b is the width and h is the height of the gasket. A gasket with dimensions of 0.005 cm in height and 0.25 cm wide was used. A computer software program, SIMPLE40, was employed to analyze the recorded video in conjunction with a standard CCD camera. Two-minute segments of the videos were analyzed at various time points and positions throughout the flow adhesion assay. An average was taken of the total number of cells that were either considered rolling or sticking to the monolayer, so that a value of cells per field was obtained and compared. ‘Rolling’ cells were considered to have velocities from 1.2–100 μm/s; ‘sticking’ cells had velocities of 0–1.2 μm/s.

### Invasion Assay

The invasion assays were performed using BD BioCoat Matrigel Invasion Chamber and BD control inserts (Becton-Dickinson, MA). The cells, as orospheres and sorted for SLe^x^ expression by flow cytometry, were re-suspended in serum-free DMEM and then added onto inserts with uncoated filter (control inserts) or onto inserts with Matrigel coated filters (Invasion chambers) at the density of 5×10^4^ cells/insert. DMEM containing 10% FBS was used as the chemoattractant. After incubation of 22 hours at 37°C, 5% CO_2_, non-invading cells on the upper side of the membrane were removed with cotton swabs and the invading cells were fixed using methanol and stained with hematoxylin. Cells that invaded were counted at 200X magnification. Each assay was performed in triplicate. Invasion was calculated as percentage of cells that invaded the Matrigel insert compared to the number of cells that migrate in the control insert.

### Statistical analysis

Statistical analyses were carried out by University of Michigan Center for Statistical Consultation and Research (CSCAR). An Independent Samples test was run using the SPSS program to analyze the significance of the differences found. P-values less than 0.05 were considered to be statistically significant. In all graphs, error bars represent the 95% confidence interval.

## SUPPLEMENTARY FIGURES AND TABLES


