# Supplementary material for: Increased fucosylation has a pivotal role in invasive and metastatic properties of head and neck cancer stem cells
Source: Oncotarget. 2014 Nov 6;6(1):71–84. doi: 10.18632/oncotarget.2698 (PMC4381579; doi:10.18632/oncotarget.2698)
Supplement: Supplementary file 1 [file oncotarget-06-071-s001.pdf]

Increased fucosylation has a pivotal role in invasive and metastatic properties of head and neck cancer stem cells

Supplementary Material

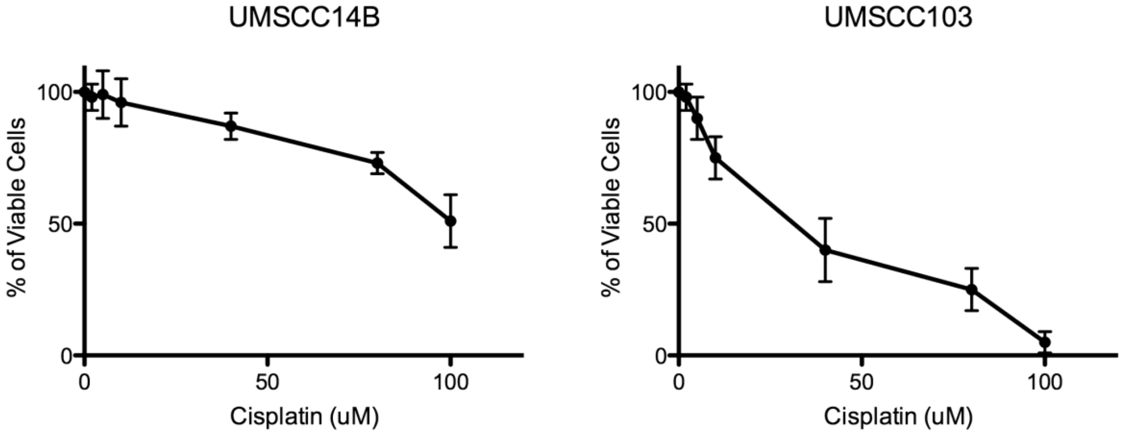

Supplementary Figure 1: Preliminary dose-response curve to cisplatin of UMSCC14B and UMSCC102 cell lines.

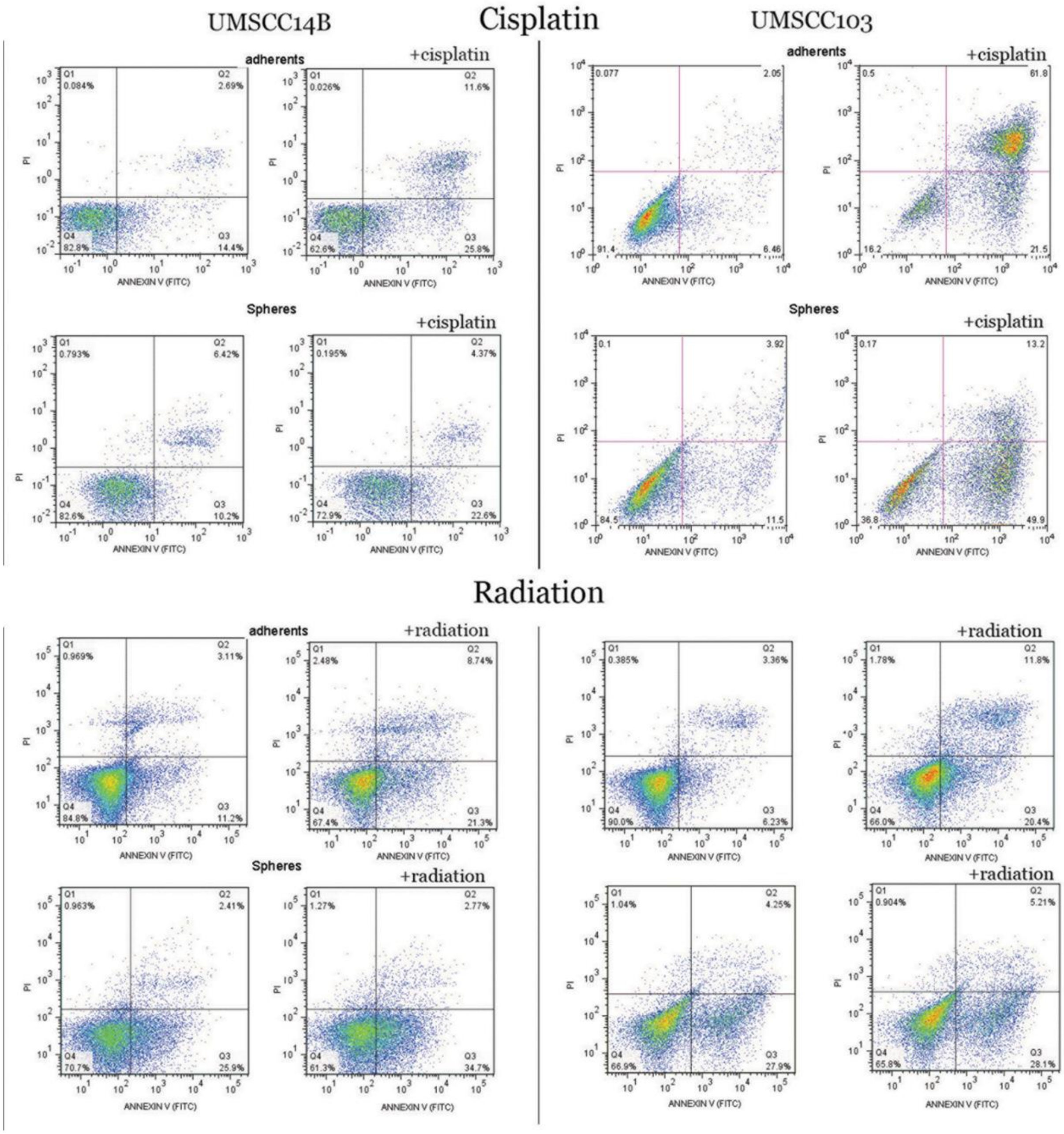

Supplementary Figure 2: Cisplatin and radiation treatments on UMSCC14B and UMSCC103 cell lines. **A**. Annexin V/PI staining on orospheres and adherent cells treated with cisplatin for 24h. Orospheres of both lines are more resistant to cisplatin. **B**. Annexin V/PI staining on spheroids and adherent cells 48h after radiation treatment. For all the cell lines considered, orospheres are more resistant to radiation.

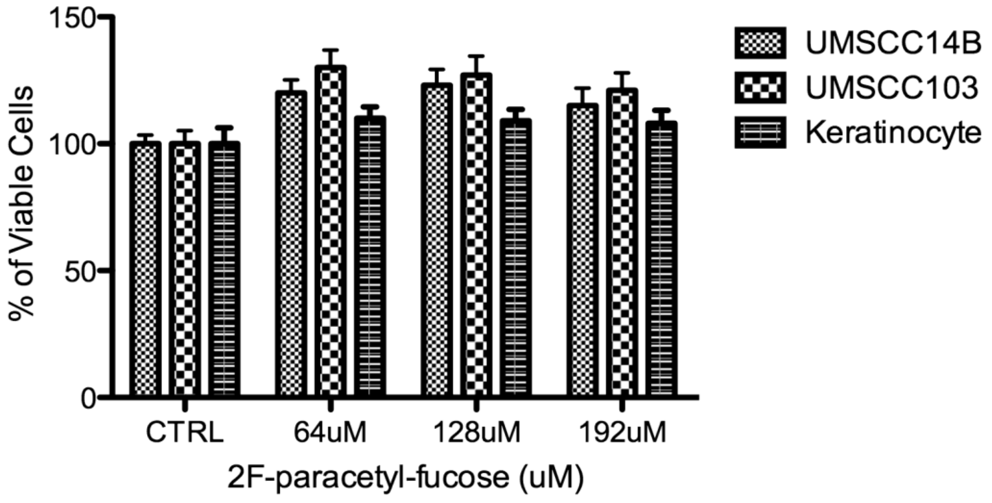

Supplementary Figure 3: Effect of 2F-paracetyl-fucose on viability of UMSCC14B, UMSCC103, and normal keratinocytes assessed by MTT assay.

Supplemetary table 1: **PCR array summary and primer sequences**

A. PCR array, fold increase orospheres vs adherent

	NCAM	ALDH	VEGF	CXCR2	MMP10	PAR6	*FXYD5*
UMSCC14B	3,7	12,5	1,7	2,5	14	1.9	2
UMSCC103	2	20	1,6	5,8	14	6,1	1,8

B. Primers sequences for RT-PCR

Gene name		5'- Sequence -3'
NANOG	Forward	CAGCTGTGTGTACTCAATGATAGATTT
	Reverse	CAACTGGCCGAAGAATAGCAATGGTGT
Oct3/4	Forward	ATGTGGTCCGAGTGTGGTTC
	Reverse	GAGACAGGGGGAAAGGCTTC
Sox2	Forward	AACCCCAAGATGCACAACTC
	Reverse	CGGGGCCGGTATTTATAATC
Fut 3	Forward	GCCGACCGCAAGGTGTAC
	Reverse	TGACTTAGGGTTGGACATGATATCC
Fut5	Forward	ACTCTGACCCATGGATCCCC
	Reverse	GTGACAGGTTCCACTGCCAT
Fut6	Forward	TTTGTGGCCAACCCTCTCTC
	Reverse	CAGGCAAGTCTTCTGGAGGG
Fut7	Forward	GATGAATAATGCTGGGCACGG
	Reverse	GTGCCAGACAAGGATGGTGA

Supplementary table 2. **Cisplatin and Radiation resistance**

Colony forming assay (rate of colony formation, % of control)

	UMSCC14B		UMSCC103	
	Adherent	Spheres	Adherent	Spheres
Radiation	8%	35%*	12%	25%*
Cisplatin	1%	10%*	4%	16%*

*p<0,05;

Cisplatin resistance (Annexin V/PI assay)

	UMSCC14B				UMSCC103			
	Adherent		Orospheres		Adherent		Orospheres	
	CTRL	*Cisplatin*	*CTRL*	*Cisplatin*	*CTRL*	*Cisplatin*	*CTRL*	*Cisplatin*
Viability	82,8%	62,6%	82,6%	72,9%	91,4%	16,2%	84,5%	36,8%
Apoptosis	17,0%	37,4%	16,6%	26,9%	8,5%	83,3%	15,4%	63,1%
Necrosis	0,08%	0,03%	0,8%	0,2%	0,08%	0,5%	0,1%	0,17%

Radiation Resistance (Annexin V/PI assay)

	UMSCC14B				UMSCC103			
	Adherent		Orospheres		Adherent		Orospheres	
	CTRL	*Radiation*	*CTRL*	*Radiation*	*CTRL*	*Radiation*	*CTRL*	*Radiation*
Viability	84,8%	67,4%	70,7%	61,3%	90,0%	66,0%	66,9%	65,8%
Apoptosis	14,3%	30,0%	28,3%	37,4%	9,6%	32,2%	32,2%	33,3%
Necrosis	1,0%	2,4%	1,0%	1,2%	0,4%	1,8%	1,0%	0,9%
